# Evaluation of time courses of agreement between minutely obtained transcutaneous blood gas data and the gold standard arterial data from spontaneously breathing Asian adults, and various subgroup analyses

**DOI:** 10.1186/s12890-020-01184-w

**Published:** 2020-05-29

**Authors:** Akira Umeda, Masahiro Ishizaka, Masamichi Tasaki, Tateki Yamane, Taiji Watanabe, Yasushi Inoue, Taichi Mochizuki, Yasumasa Okada, Sarah Kesler

**Affiliations:** 1 Departments of Internal Medicine, International University of Health and Welfare (IUHW) Shioya Hospital, Tomita 77, Yaita-City, Tochigi 329-2145 Japan; 2 Departments of Rehabilitation, International University of Health and Welfare (IUHW) Shioya Hospital, Yaita-City, Japan; 3 Department of Internal Medicine, National Hospital Organization Murayama Medical Center, Musashimurayama-City, Japan; 4 Intensive Care Unit, University of Minnesota, Minneapolis, MN USA

**Keywords:** Transcutaneous, Blood gas, Bland-Altman analysis, Non-invasive, Time course, Agreement, Subgroup analysis

## Abstract

**Background:**

Usual clinical practice for arterial blood gas analysis (BGA) in conscious patients involves a one-time arterial puncture to be performed after a resting period of 20–30 min. The aim of this study was to evaluate the use of transcutaneous BGA for estimating this gold standard arterial BGA.

**Methods:**

Spontaneously breathing Asian adults (healthy volunteers and respiratory patients) were enrolled (*n* = 295). Transcutaneous PO_2_ (PtcO_2_) and PCO_2_ (PtcCO_2_) were monitored using a transcutaneous monitor (TCM4, Radiometer Medical AsP, Denmark) with sensors placed on the chest, forearm, earlobe or forehead. Transcutaneous BGA at 1-min intervals was compared with arterial BGA at 30 min. Reasonable steps to find severe hypercapnia with PaCO_2_ > 50 mmHg were evaluated.

**Results:**

Sensors on the chest and forearm were equally preferred and used because of small biases (*n* = 272). The average PCO_2_ bias was close to 0 mmHg at 4 min, and was almost constant (4–5 mmHg) with PtcCO_2_ being higher than PaCO_2_ at ≥8 min. The limit of agreement for PCO_2_ narrowed over time: ± 13.6 mmHg at 4 min, ± 7.5 mmHg at 12–13 min, and ± 6.3 mmHg at 30 min. The limit of agreement for PO_2_ also narrowed over time (± 23.1 mmHg at 30 min). Subgroup analyses showed that the PaCO_2_ and PaO_2_ levels, gender, and younger age significantly affected the biases. All hypercapnia subjects with PaCO_2_ > 50 mmHg (*n* = 13) showed PtcCO_2_ ≥ 50 mmHg for until 12 min.

**Conclusions:**

Although PtcCO_2_ is useful, it cannot completely replace PaCO_2_ because PCO_2_ occasionally showed large bias. On the other hand, the prediction of PaO_2_ using PtcO_2_ was unrealistic in Asian adults. PtcCO_2_ ≥ 50 mmHg for until 12 min can be used as a screening tool for severe hypercapnia with PaCO_2_ > 50 mmHg.

## Background

The partial pressure of blood gases can be estimated through measurement of dissolved gases that diffuse to the skin surface [1, 2]. Measurement of transcutaneous PO_2_ (PtcO_2_) and PCO_2_ (PtcCO_2_) requires local heating of the skin, which dilates vessels and increases arterial blood supply to the skin capillary bed under the sensor, resulting in accelerated gas diffusion [3, 4]. In clinical practice, this method is widely used to assess pulmonary gas exchange function in infants and children, and in adults with acute or chronic respiratory failure [5–7]. It may also be applied to monitoring the condition of patients on mechanical ventilation and managing limb ischemia [8–10].

Although previous studies have investigated the relationship between PtcCO_2_ and PaCO_2_ over time, the time courses of transcutaneous data for the estimation of arterial blood gas analysis (BGA) are not well characterized [11–13]. Various factors may influence the time course of agreement including the response speed of the electromechanical gas measuring system, the speed of skin heating, and the time to equilibration of gases [3, 13–15]. This information would allow physicians to choose a convenient (early) time-point for transcutaneous BGA for the estimation of arterial BGA and an optimal time-point for increased accuracy.

A previous study suggested that the correlation between arterial BGA and transcutaneous BGA data via sensors on the chest is stronger than that observed via sensors on the arm; however, this report involved only anesthetized adult patients [16]. The most commonly recommended sensor location, according to the guidelines established by the American Association for Respiratory Care, is the upper chest followed by the lateral side of the abdomen, chest, buttock, inside of the upper thigh, forearm, the zygomatic bone, the ear lobe, cheek, or the forehead in neonates and small pediatric patients [9]. In the beginning, we compared data obtained from sensors placed on the chest, forearm, earlobe, and forehead in spontaneously breathing adults. In the early stage of the study, we decided to use only a chest or forearm sensor (data are shown later).

Arterial blood samples are drawn with the patient being in a steady state [17]. The usual clinical practice for arterial BGA in fully conscious patients involves a single arterial puncture performed after a waiting period of 20–30 min [17, 18]. The procedure of arterial puncture may cause pain and hyperventilation, thereby altering subsequent arterial BGA data due to respiratory alkalosis [17]. In mechanically ventilated patients, the stability after a change in F_I_O_2_ is reached between 10 and 30 min depending on the physiological and pathophysiological conditions of the patient [19]. Therefore, in the present study, the arterial BGA data with one-time arterial puncture after a waiting (resting) period of 30 min in the supine position was defined as the gold standard blood gas data.

We evaluated the transcutaneous BGA data at 1-min intervals comparing the final goal of arterial BGA at 30 min. This novel approach will answer the following questions: “From which time point are the transcutaneous BGA data meaningful?” and “How accurately are the current transcutaneous BGA data predicting arterial BGA?” In addition, the results of the subgroup analyses which may help to understand transcutaneous BGA, are shown. Finally, we discuss the most important subgroup (i.e., severe hypercapnia with PaCO_2_ > 50 mmHg) and recommend a reasonable time-saving step for the accurate diagnosis of these patients.

## Methods

### Subjects and study procedures

The study was approved by the Ethics Committee of the International University of Health and Welfare (IUHW, approval number 13-B-109). All subjects provided written consent prior to participating in this study. All subjects were adults, aged ≥20 years. Both healthy volunteers and patients who visited the Department of Respiratory Medicine, IUHW Shioya Hospital were invited to participate in the study. Measurements were performed in the supine position at room temperature (24–25 °C). Transcutaneous BGA data from 1 to 30 min and arterial BGA data at 30 min were obtained, and compared through Bland–Altman analysis [20]. Monitoring of percutaneous oxygen saturation (SpO_2_) with a pulse oximeter (PULSOX-C; KONICA MINOLTA, Osaka, Japan) was performed to confirm that SpO_2_ data from each subject were constant during the study (from sensor fixation to arterial blood sampling).

### Transcutaneous BGA

PtcO_2_ and PtcCO_2_ were measured with a transcutaneous gas monitoring system (TCM4 with tcSensor 84 for neonatal, pediatric and adult patients; Radiometer Medical AsP, Copenhagen, Denmark), using the principles of the Clark and Severinghaus electrodes [21]. Calibration was achieved within 5–6 min. Following membrane change, the calibration required approximately 10 min. The temperature of the skin probe was set at 44 °C, as previously recommended [3, 8, 9]. Measurements were performed in the supine position with the skin probe position on (i) the upper chest wall (left or right second intercostal space in the midclavicular line), (ii) the inside of the forearm (upper third of the inner surface of the left or the right forearm), (iii) the earlobe, or (iv) the forehead. The sensor location was randomized and the duration of measurements was ≥30 min. Prior to sensor fixation, the skin and electrode were thoroughly cleaned, and an adhesive ring with two drops of contact gel was applied, according to the instructions provided by the manufacturer. Chest hair was avoided, and depilation from the thoracic skin was not necessary. After sensor fixation, PtcO_2_ and PtcCO_2_ were recorded at 1-min intervals. Prior to sensor fixation, the sensor detected the atmospheric PO_2_ and PCO_2_. The electromechanical response of the TCM4 device, as shown by breathing on the sensor measuring atmospheric PO_2_ and PCO_2_, elicited a decrease in PO_2_ within 5 s and an increase in PCO_2_ within 15 s.

### Arterial BGA

Arterial BGA was performed at 30 min after sensor fixation in the supine position. Femoral arterial blood (0.5–1.0 ml) was drawn with a 22-gauge needle attached to a heparinized syringe. Samples were immediately analyzed with a blood gas analyzer (Rapidlab 1265; Siemens Healthcare Diagnostics, Sudbury, United Kingdom).

### Subgroup analysis

The effects of gender, age, PaCO_2_ level and PaO_2_ level on the agreement data were evaluated. The transcutaneous data obtained via the sensors on the chest or forearm were used. The subjects with a PaCO_2_ level within the normal range (35–45 mmHg) were compared with those having different levels of hypocapnia or hypercapnia [17]. The subjects with a PaO_2_ level with the normal range (80–100 mmHg) were compared with those having hypoxemia or hyperoxemia [17].

### Data analysis

Data are expressed as means ± standard deviation (SD), unless otherwise indicated. Concordance of arterial (at 30 min) and transcutaneous (between 1 and 30 min) blood gas data were investigated by Bland–Altman analysis. Therefore, 30 Bland–Altman analyses were performed. Analysis of variance with Tukey’s correction or unpaired t-test (two-tailed) was used for the comparison at 30 min. The Excel Statistics software, 2010 version (Social Survey Research Information Co., Ltd., Tokyo, Japan) was used. *P* < 0.05 denoted a statistically significant difference. Data were accumulated from the usual clinical practice at IUHW Shioya Hospital. When the number of subjects using chest or forearm sensor was 163 (males: 107, females: 56), PtcCO_2_ at 30 min was markedly larger than PaCO_2_ at 30 min (*P* = 3.8 × 10^− 42^). In the subgroup analysis, the absolute values of PO_2_ bias were larger in males than in females with *P* = 0.050. We calculated the necessary sample size to evaluate this interesting effect of gender. The expected effect size was 4.0 mmHg (SD: 12.1 mmHg); therefore, the standardized effect size was 0.33 [22]. It was considered that approximately 100 samples per group were necessary for an 80% power to detect significance at the 10% level (two-sided) in a t-test. The number of subjects using a chest or forearm sensor increased to 272 (males: 168, females: 104). The *P*-value decreased to < 0.01 and the effect of gender was confirmed (data are shown later).

## Results

The tolerance of local heating for electrode attachment was good. There were no signs of skin irritation or erythema at the end of the monitoring.

### Study population

**Fig. 1:**
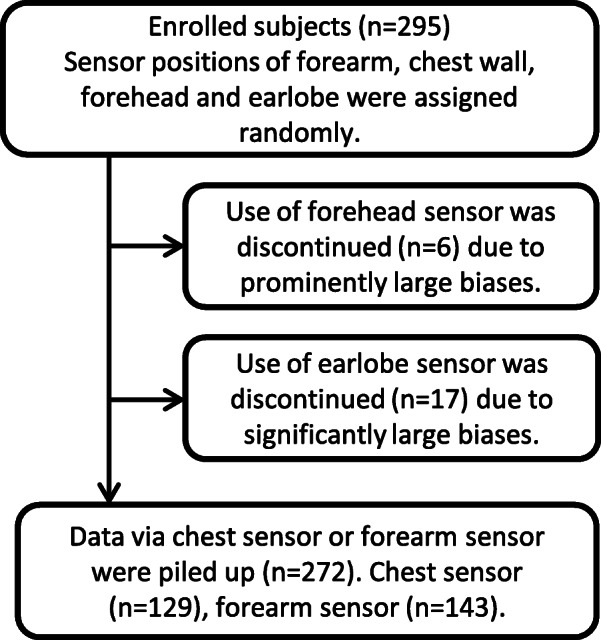
Process of data collection via the chest or forearm sensors

A total of 295 spontaneously breathing Asian adults (184 males, 111 females; mean age: 73.6 ± 14.5 years), comprising 10 healthy volunteers and 285 patients with various lung diseases, were enrolled from August 2015 to August 2019. The breakdown of lung diseases is shown in Supplementary Table S1 (Additional file 1). Forty-nine subjects received oxygen therapy. These subjects were studied while receiving supplemental O_2_. Initially, four sensor positions were randomly used. However, shortly after the study was initiated, the use of the forehead as a sensor location was discontinued because of prominently large biases (Fig. 1). The use of the earlobe as sensor location was also discontinued because of large biases (data shown later in this article). Age, gender, body mass index and diagnosed diseases were almost equally distributed among the four groups (one group per probe; Additional file 1, Supplementary Table S1). Monitored SpO_2_ was stable and constant (i.e., ≤ 2% change during the 30 min observation period in any subject).

### Agreement at 30 min

**Fig. 2:**
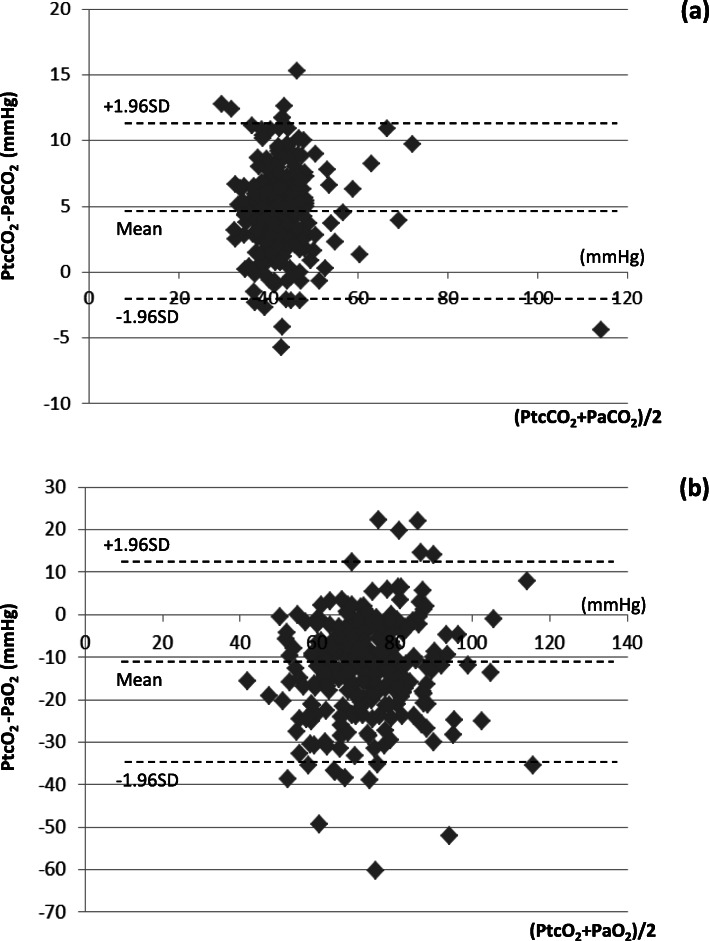
Blood gas data at 30 min (transcutaneous data were obtained via the chest or forearm sensors, *n* = 272). **a** Concordance between PaCO_2_ and PtcCO_2_ data at 30 min. The bias of PCO_2_ was 4.7 mmHg, with PtcCO_2_ being higher than PaCO_2_ and 95% limits of agreement of ±6.3 mmHg. **b** Concordance between PaO_2_ and PtcO_2_ data at 30 min. The bias of PO_2_ was 12.2 mmHg, with PaO_2_ being higher than PtcO_2_ and 95% limits of agreement of ±23.1 mmHg

The concordances between transcutaneous BGA and arterial BGA data at 30 min are shown in Fig. 2. Transcutaneous data obtained via the chest or forearm sensors were used (*n* = 272). The bias of PCO_2_ was 4.7 mmHg, with PtcCO_2_ being higher than PaCO_2_, and the 95% limits of agreement were ± 6.3 mmHg (Fig. 2a). The bias of PO_2_ was 12.2 mmHg, with PaO_2_ being higher than PtcO_2_, and the 95% limits of agreement were ± 23.1 mmHg (Fig. 2b).

### Time courses of agreement

**Fig. 3:**
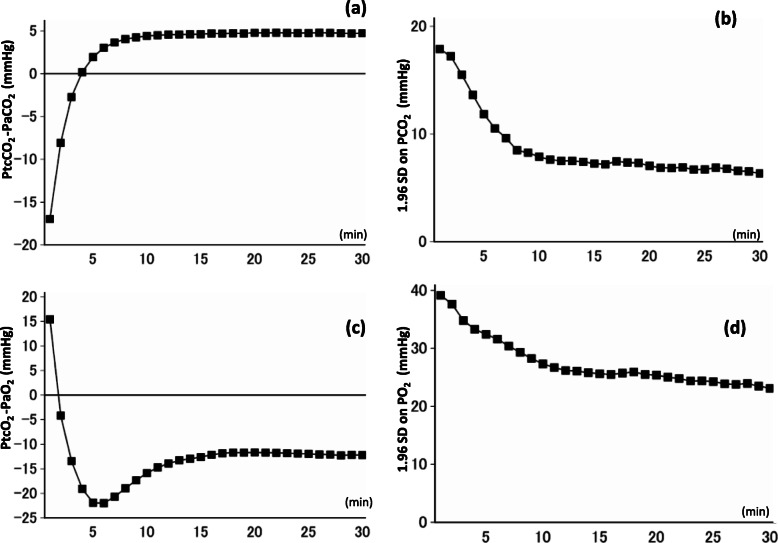
Time courses of Bland-Altman analysis indices: the bias and 95% limits of agreement (transcutaneous data were obtained via the chest or forearm sensors, *n* = 272). Average data from 272 subjects are shown. **a** Bias and **b** 1.96SD on PCO_2_; **c** bias and **d** 1.96SD on PO_2_. The bias on PCO_2_ was 0.2 mmHg at 4 min (**a**), and subsequently increased to a plateau of 4.1–4.8 mmHg, which was almost stable between 8 and 30 min. 1.96SD on PCO_2_ declined over time, with the minimum (6.3 mmHg) observed at 30 min; however, it remained almost constant (6.3–7.5 mmHg) between 12 and 30 min, (**b**). The bias on PO_2_ initially decreased, passed the nadir at 5–6 min, and plateaued between 16 and 30 min (**c**). 1.96SD on PO_2_ decreased over time, with the minimum (23.1 mmHg) observed at 30 min (**d**)

The time courses of the two indices used in the Bland–Altman analysis (the bias and the 95% limits of agreement) are shown in Fig. 3. This was a series of 30 Bland–Altman analyses in which we compared 1–30 min transcutaneous data with min-30 arterial BGA data. Transcutaneous data obtained via the chest or forearm sensors were used (*n* = 272). The bias of PCO_2_ was 0.2 mmHg at 4 min (Fig. 3a), and the 95% limits of agreement (± 1.96SD) were ± 13.6 mmHg (Fig. 3b). This 1.96SD for PCO_2_ was initially reduced; however, it was similar to that obtained between 12 and 30 min (6.3–7.5 mmHg). At 8 min or later, the bias was 4.1–4.8 mmHg, with PtcCO_2_ being higher than PaCO_2_. In contrast, the absolute value of bias of PO_2_ was lowest (almost 0 mmHg) at 1–2 min (Fig. 3c), and the 1.96SD was reduced over time, with the closest agreement observed at 30 min (± 23.1 mmHg) (Fig. 3d).

### Differences in agreement among four sensor locations

**Fig. 4:**
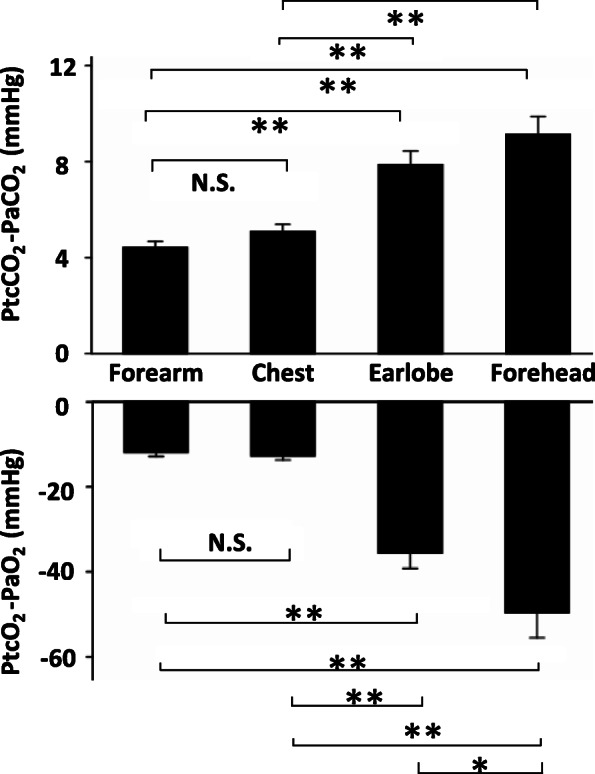
Comparison of bias at 30 min among four sensor locations. The absolute values of bias obtained via forearm (*n* = 143) and chest (*n* = 129) sensors were equivalent, and were significantly lower than those obtained via the earlobe (*n* = 17) or forehead (*n* = 6) sensors for both PCO_2_ and PO_2_. The absolute values of PO_2_ bias obtained via the earlobe sensor were significantly lower than those obtained via the forehead. ANOVA with Tukey’s pot-hoc test, *: *P* < 0.05, **: *P* < 0.01, Bars: SEM

The time courses of the agreement data were compared among the four sensor locations (Additional file 2, Supplementary Fig. S1). At 30 min, the absolute values of bias obtained via the forearm and chest sensors were equivalent, and significantly lower than those obtained via the earlobe or forehead sensors for both PCO_2_ and PO_2_ (Fig. 4). At 30 min, the absolute values of PO_2_ bias obtained via the earlobe sensor were significantly lower than those obtained via the forehead sensor. There was no significant difference in arterial BGA data or pulse oximeter data among the four different sensor location groups (Additional file 1, Supplementary Table S2). On the other hand, PtcO_2_ data obtained via the earlobe or forehead sensor at 30 min were significantly different from those obtained via the forearm or chest sensor at 30 min.

### Subgroup analyses

#### Effects of gender and age

**Fig. 5:**
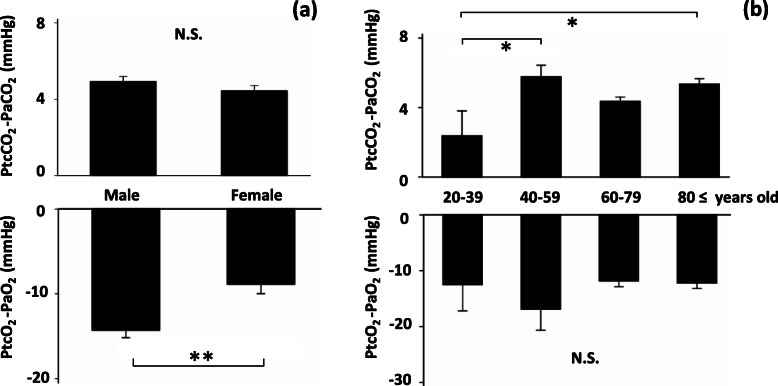
Subgroup analyses on gender and age at 30 min. Transcutaneous data obtained via the chest or forearm sensors were used (*n* = 272). Bars: SEM, *: *P* < 0.05, **: *P* < 0.01. **a** Comparison of biases between males (*n* = 168) and females (*n* = 104). Gender had no significant effects on PCO_2_ bias. However, the absolute values of male PO_2_ bias observed were significantly larger than those of female PO_2_ bias (Unpaired t-test). **b** Comparison of biases among 4 age groups: 20–39 years (*n* = 11), 40–59 years (*n* = 12), 60–79 years (*n* = 138), and ≥ 80 years (*n* = 111). PCO_2_ biases in young adults (20–39 years) were significantly lower than in the 2 groups of 40–59 years and ≥ 80 years. However, there were no significant differences in PO_2_ bias among the four groups (ANOVA with Tukey’s pot-hoc test)

The effects of gender on the time courses were examined: male (*n* = 168), female (*n* = 104, Additional file 2, Supplementary Fig. S2). At 30 min, the absolute values of PO_2_ bias in males were significantly larger than those recorded in females (*P* < 0.01; Fig. 5a). The effects of age on the time courses were examined in the following four groups: 20–39 years (*n* = 11), 40–59 years (*n* = 12), 60–79 years (*n* = 138), and ≥ 80 years (*n* = 111) (Additional file 2, Supplementary Fig. S3). At 30 min, the PCO_2_ biases in young adults (20–39 years old) were significantly lower than those observed in the 40–59 year-old group and ≥ 80 year-old group (both *P* < 0.05; Fig. 5b).

#### Effects of PaCO_2_ and PaO_2_ levels

**Fig. 6:**
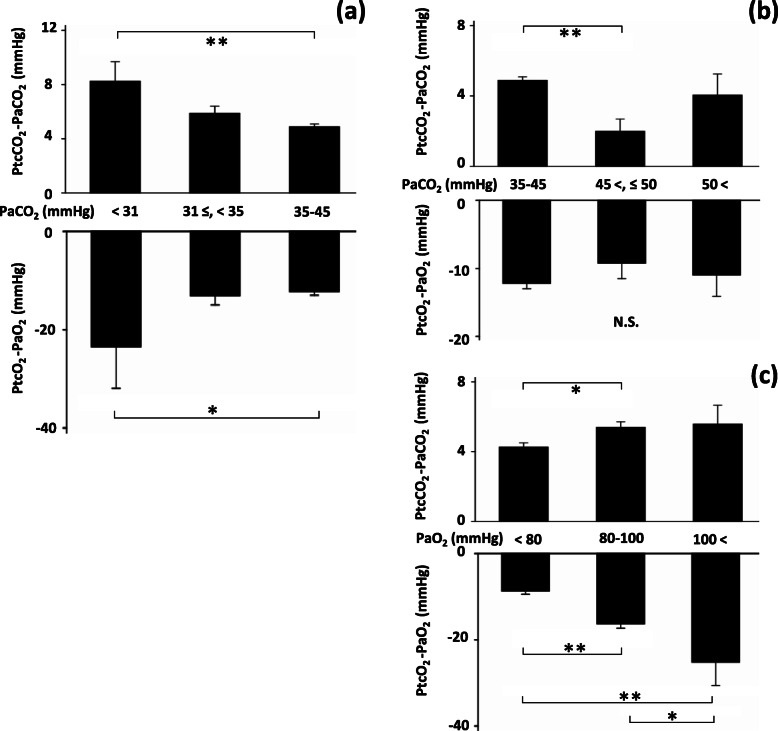
Subgroup analyses on PaCO_2_ level and PaO_2_ level at 30 min. Transcutaneous data obtained via the chest or forearm sensors were used (*n* = 272). Bars: SEM, *: *P* < 0.05, **: *P* < 0.01, ANOVA with Tukey’s pot-hoc test. **a** Comparison of biases among severe hypocapnia (PaCO_2_ < 31 mmHg, *n* = 7), mild hypocapnia (31 mmHg ≤ PaCO_2_ < 35 mmHg, *n* = 24), and normal range (35 mmHg ≤ PaCO_2_ ≤ 45 mmHg, *n* = 202). The absolute values of bias observed in the severe hypocapnia group were significantly larger than those observed in the normal range group for both PCO_2_ and PO_2_. This effect of hypocapnia seemed to be intensity-dependent. **b** Comparison of biases among normal range (35 mmHg ≤ PaCO_2_ ≤ 45 mmHg, *n* = 202), mild hypercapnia (45 mmHg < PaCO_2_ ≤ 50 mmHg, *n* = 26), and severe hypercapnia (50 mmHg < PaCO_2_, *n* = 13). PCO_2_ biases in mild hypercapnia were significantly smaller than in normal range group. **c** Comparison of biases among hypoxemia (PaO_2_ < 80 mmHg, *n* = 158), normal range (80 mmHg ≤ PaO_2_ ≤ 100 mmHg, *n* = 102), and hyperoxemia (100 mmHg < PaO_2_, *n* = 12). The absolute values of bias observed in the hypoxemia group were significantly smaller than those observed in the normal range group for both PCO_2_ and PO_2_. The absolute values of PO_2_ bias observed in the hyperoxemia group were significantly larger than those observed in the normal range group. These effects on PO_2_ biases seemed to be PaO_2_ level-dependent

The effects of hypocapnia on the time courses were examined in the following two levels of PaCO_2_: PaCO_2_ < 31 mmHg group (severe hypocapnia, *n* = 7) and 31 ≤ PaCO_2_ < 35 mmHg group (mild hypocapnia, *n* = 24; Additional file 2, Supplementary Fig. S4). At 30 min, the absolute values of bias in the severe hypocapnia group were higher than those noted in the normal PaCO_2_ group (*n* = 202) for both PCO_2_ (*P* < 0.01) and PO_2_ (*P* < 0.05; Fig. 6a). This effect of hypocapnia on the PCO_2_ bias was dependent on severity. The effects of hypercapnia on the time courses were examined in the following two levels of PaCO_2_: 45 < PaCO_2_ ≤ 50 mmHg group (mild hypercapnia, *n* = 26) and > 50 mmHg group (severe hypercapnia, *n* = 13; Additional file 2, Supplementary Fig. S5). At 30 min, the PCO_2_ bias in the mild hypercapnia group was significantly lower than that observed in the normal PaCO_2_ group (*P* < 0.01; Fig. 6b). The effects of the PaO_2_ level on the time courses were similarly examined (Additional file 2, Supplementary Fig. S6). At 30 min, the absolute values of bias in the hypoxemia group (*n* = 158) were lower than those measured in the normal PaO_2_ group (*n* = 102) for both PCO_2_ (*P* < 0.05) and PO_2_ (*P* < 0.01; Fig. 6c). At 30 min, the absolute values of PO_2_ bias in the hyperoxemia group (*n* = 12) were larger than those recorded in the normal PaO_2_ group (*P* < 0.05; Fig. 6c).

### Precise observation of subjects with hypercapnia PaCO_2_ > 50 mmHg

Data and profiles of subjects with hypercapnia (PaCO_2_ > 50 mmHg, *n* = 13) are shown in Table 1. Most of these subjects (85%) were receiving O_2_ therapy. At 4 min, PtcCO_2_ ≥ 50 mmHg was observed in 69% of these subjects. This ratio increased over time; at 12 min, all these subjects showed PtcCO_2_ ≥ 50 mmHg. At 13 min, all these subjects showed PtcCO_2_ ≥ 51 mmHg. Subjects with severe hypercapnia appeared to have more diseases (e.g., circulatory failure) than those with mild hypercapnia.

**Table 1:** Data and profiles of subjects with hypercapnia > 50 mmHg (*n* = 13)

No.	Age	O_2_ therapy (L/min)	PaCO_2_ (mmHg)	PtcCO_2_ (mmHg)						PaO_2_ (mmHg)	PtcO_2_ (mmHg)	Characteristics	Blood pressure (mmHg)	Body temperature	Circulatory failure	Edema
			at 30 min	at 4 min	at 10 min	at 11 min	at 12 min	at 13 min	at 30 min	at 30 min	at 30 min					
1	76	2 (nasal)	50.4	53	54	55	56	56	57	111.6	98	ACO	130/78	Normal (?)	–	–
2	82	3 (nasal)	51.7	42	49	50	50	51	51	93.3	58	ACO, pneumonia	119/53	35.3 °C	–	–
3	84	1.5 (nasal)	52.3	51	57	57	57	56	56	77.3	75	ACO	160/75	Normal (?)	–	–
4	81	1 (nasal)	52.7	52	55	55	54	54	53	79	53	COPD (E), ILD	128/59	Normal (?)	–	–
5	62	Room air	53.7	46	55	56	56	56	56	55	55	OHS	139/75	Normal (?)	–	–
6	65	3 (nasal)	54.5	40	55	56	56	57	59	60.7	60	OHS, HF	112/59	36.2 °C	+	+
7	89	4 (face mask)	55.7	33	49	49	51	52	62	50.5	50	COPD (E)	126/83	Normal (?)	–	–
8	88	Room air	58.8	59	66	66	67	67	67	80.7	72	COPD (E), HF, ILD	81/40	Normal (?)	+	±
9	79	1.5 (nasal)	59.7	54	62	62	62	61	61	79.8	76	COPD (E)	135/88	Normal (?)	–	–
10	85	6 (face mask)	61.1	61	71	71	68	67	72	87.9	64	OHS, airway infection	139/73	37.3 °C	Suspected	–
11	81	1.25 (nasal)	67.1	57	72	72	72	71	71	49.7	34	COPD (E)	119/81	Normal (?)	Suspected	±
12	70	3 (face mask)	67.3	57	80	81	81	81	77	81	77	ACO, HF	135/61	36.7 °C	+	–
13	82	5 (face mask)	116.4	50	88	91	92	92	112	74.9	67	COPD (E), MOF	90/45	38.5 °C	+	++

No skin eruption was seen in any subjects. It was difficult to predict hypercapnia by underlined PtcCO_2_ data. All hypercapnia subjects with PaCO_2_ > 50 mmHg showed PtcCO_2_ ≥ 50 mmHg for until 12 min. All the transcutaneous blood gas analysis data were obtained via chest sensor or forearm sensor. *ACO* Asthma-chronic obstructive pulmonary disease (COPD) overlap, *COPD* Chronic obstructive pulmonary disease, *E* Emphysema, *HF* Heart failure, *ILD* Interstitial lung disease, *MOF* Multiple organ failure, *OHS* Obesity hypoventilation syndrome

## Discussion

By comparing the agreement between minutely obtained transcutaneous BGA data and the final answer data of arterial BGA at 30 min, we obtained the following findings. Firstly, the sensors placed on the chest and forearm are equally preferred. Secondly, the method to predict PaCO_2_ at 30 min is to initially measure PtcCO_2_ at 4 min without bias, and observe PtcCO_2_ at 8 min or later considering a bias of 4–5 mmHg. Thirdly, although PtcCO_2_ is useful, it cannot completely replace the actual levels of PaCO_2_ due to occasional large PCO_2_ bias. Fourthly, the subgroup analyses showed that gender, younger age, PaCO_2_ levels, and PaO_2_ levels affected PO_2_ and/or PCO_2_ biases. Fifthly, a reasonable step to reach accurate diagnosis of PaCO_2_ > 50 mmHg using transcutaneous BGA data was recommended. Finally, we showed that the prediction of PaO_2_ by PtcO_2_ was unrealistic in Asian adults.

Previously, it was reported that the 1.96SD between venous PCO_2_ and PaCO_2_ was 15.0 mmHg [23]. Venous PCO_2_ is occasionally used as a surrogate with a bias of 5 mmHg. Our approach enabled to answer the question of “From which time point are the PtcCO_2_ data meaningful?” The answer is “From 4 min.”, because the limits of agreement between PaCO_2_ and PtcCO_2_ at 4 min or later were ± 13.6 mmHg or narrower. Of note, they were narrower than the limit of agreement (± 15.0 mmHg) between PaCO_2_ and venous PCO_2_. By waiting longer, we can obtain more accurate PtcCO_2_ data for the estimation of PaCO_2_. Several studies have indicated that PtcCO_2_ is more accurate than end-tidal PCO_2_ as a surrogate measure of PaCO_2_ [24–28]. While 1.96SD data between end-tidal PCO_2_ and PaCO_2_ ranged from 6.9 to 14.4 mmHg, 1.96SD data between PtcCO_2_ and PaCO_2_ ranged from 4.6 to 10.4 mmHg. The PtcCO_2_ data at 12–13 min or later were within the acceptable clinical range of agreement for PtcCO_2_ (± 7.5 mmHg) recommended in the guideline of the American Association for Respiratory Care [9].

As a whole, the prediction of PaCO_2_ is possible. It involves initial measurement of PtcCO_2_ at 4 min without bias, and observation of PtcCO_2_ at 8 min or later considering a bias of 4–5 mmHg. In a steady state, PtcCO_2_ is higher than PaCO_2_ because the former is an epidermal parameter which does not exclusively reflect arterial blood, and CO_2_ is produced by living epidermal cells [7, 29, 30].

The 1.96SD between PtcO_2_ and PaO_2_ displayed a continual decline without an obvious plateau at 30 min. Even the minimal limit of agreement of ±24.0 mmHg at 30 min is not negligible in clinical practice. Therefore, PtcO_2_ is not an appropriate substitute for PaO_2_. Kesten et al. reported that the 90% response speed of PtcCO_2_ in this system was approximately three times faster than that of PtcO_2_ [14]. The Krogh’s constants of diffusion for O_2_ and CO_2_ in water and aqueous tissues may be important to understand the difference between these gases [15]. In water and aqueous tissues, the Krogh’s constant of diffusion for CO_2_ has been reported to be 20–25 times higher than that for O_2_.

A change in the baseline level with time is termed “drift” [31]. The calibration was performed prior to measuring each subject according to the protocol provided by the manufacturer. The duration of the measurement was only 30–40 min per subject; therefore, the “drift” effect was considered negligible.

A few previous studies have investigated the relationship between PtcCO_2_ and PaCO_2_ over time [11–13]. Fuke et al. compared PaCO_2_ via an arterial catheter and PtcCO_2_ over time (*n* = 6), yielding evaluations of individual agreements [11]. Excellent agreement over time was shown in three subjects. Both Cuvelier et al. [12] and Storre et al. [13] compared PaCO_2_ via an arterial catheter and PtcCO_2_ over time (*n* = 12 and *n* = 10, respectively), demonstrating parallel changes without any description of concordance over time. The present noninvasive study without arterial catheterization is in line with the actual clinical practice for spontaneously breathing patients, and provides data from a larger sample of subjects compared with previous studies [11–13]. The subgroup analyses revealed that gender and younger age affected the biases. Further investigation is necessary to confirm this observation. The absolute values of biases (for both PCO_2_ and PO_2_) were larger in the PaCO_2_ < 31 mmHg group than in the normal group. Arterial vasoconstriction by hyperventilation may be involved in this phenomenon [23]. If PaCO_2_ is low, the PCO_2_ bias may increase and underestimation of hyperventilation might occur. However, Bendjelid et al. reported that the PaCO_2_ level did not affect the PCO_2_ bias (*n* = 55, Caucasians 85%) [32]. The absolute values of biases (for both PCO_2_ and PO_2_) were smaller in the hypoxemia group than in the normal group. Hypoxic vasodilation may be involved in this phenomenon [33].

Another limitation of the study is that arterial BGA was performed only at 30 min. However, it is worth performing Bland–Altman analysis for the comparison of the single time point arterial BGA data with the minutely obtained transcutaneous data. All gas data were collected during a short period (30–40 min) in the steady state, which was validated by observations that SpO_2_ data were almost constant in each subject from the sensor fixation to the arterial blood sampling procedure. The effect of changing body position (e.g., from sitting to supine position) on PaCO_2_ has been reported to be smaller than that exerted on PaO_2_ [34, 35]. Bland and Altman compared data from two different peak flow meters which cannot be performed simultaneously [20].

Correct diagnosis of severe hypercapnia with PaCO_2_ > 50 mmHg is important to avoid CO_2_ narcosis. This technology of TCM4 with a Severinghaus electrode is useful in identifying subjects with PaCO_2_ > 50 mmHg. By performing arterial BGA after detecting PtcCO_2_ ≥ 50 mmHg during an observation for 12 min, PaCO_2_ > 50 mmHg can be accurately measured (without exceptions at least in our 13 subjects). We recommend this reasonable step for the efficient use of PtcCO_2_ data.

## Conclusions

We compared the agreement between minutely obtained transcutaneous BGA data and the final answer data of arterial BGA at 30 min. The use of sensors on the chest and forearm is equally recommended. Although PtcCO_2_ is useful and can be used as a screening tool for severe hypercapnia, it cannot completely replace PaCO_2_. On the other hand, the prediction of PaO_2_ by PtcO_2_ was unrealistic in Asian adults. Consideration of gender, age, PaCO_2_ levels, and PaO_2_ levels may assist in improving the accuracy of estimation. Further investigations are needed to clarify the mechanisms of these factors that influence the biases. This approach may be of potential use to better understand transcutaneous BGA.

## Supplementary information

**Additional file 1: Supplementary Table S1.** Comparison of subjects among the four groups of different sensor locations. **Supplementary Table S2.** Comparison of blood gas data among the four groups of different sensor locations.

**Additional file 2: Supplementary Fig. S1.** Comparison of the time course data among the four locations of sensors. Average data are shown. Blue line: forearm (*n* = 143), red line: chest (*n* = 129), green line: earlobe (*n* = 17), and purple line: forehead (*n* = 6). Trajectories of (**a**) bias and (**b**) 1.96SD on PCO_2_. Trajectories of (**c**) bias and (**d**) 1.96SD on PO_2_. (**a**): Compared with the forearm or chest sensors, the earlobe or forehead sensors yielded larger bias for PCO_2_ at 4 min or later. (**b**): 1.96SD on PCO_2_ was similar among the four locations. (**c**): The forearm and chest sensors showed almost the same time course of bias for PO_2_. The earlobe sensor yielded larger absolute values of bias, whereas the forehead sensor yielded much larger absolute values of bias. (**d**): 1.96SD of the forehead sensor was larger than that of the forearm or chest sensors. **Supplementary Fig. S2.** Comparison of the time course data (males vs. females, *n* = 272). Transcutaneous data obtained via the chest or forearm sensors were used. Average data are shown. Blue line: males (*n* = 168), red line: females (*n* = 104). Trajectories of (**a**) bias and (**b**) 1.96SD on PCO_2_. Trajectories of (**c**) bias and (**d**) 1.96SD on PO_2_. PCO_2_ bias was similar between the two groups (**a**). The 1.96SD of females on PCO_2_ was slightly lower than male (**b**). The absolute values of female PO_2_ bias was lower than that of males (**c**). 1.96SD on PO_2_ was not affected by gender (**d**). **Supplementary Fig. S3.** Comparison of the time course data (among four age groups, *n* = 272). Transcutaneous data obtained via the chest or forearm sensors were used. Average data are shown. Blue line: 20–39 years (*n* = 11), red line: 40–59 years (*n* = 12), green line: 60–79 years (*n* = 138), purple line: ≥ 80 (*n* = 111). Trajectories of (**a**) bias and (**b**) 1.96SD on PCO_2_. Trajectories of (**c**) bias and (**d**) 1.96SD on PO_2_. Crossing the 0 line at approximately 5 min (later than in the other three groups), PCO_2_ biases in young adults (20–39 years) was slightly lower than those of the other three groups (**a**). The 1.96SD of young adults (20–39 years) on PCO_2_ was slightly higher than that of the other three groups at 21 min or later (**b**). The absolute values of PO_2_ bias in 40–59 years group seemed a little larger than those of the other three groups at 13 min or later. (**c**). The 1.96SD of young adults (20–39 years) on PO_2_ was slightly higher than that of the other three groups at 11 min or later (**d**). **Supplementary Fig. S4.** Comparison of the time course data to evaluate effects of hypocapnia. Transcutaneous data obtained via the chest or forearm sensors were used. Average data are shown. Blue line: severe hypocapnia (PaCO_2_ < 31 mmHg, *n* = 7), red line: mild hypocapnia (31 mmHg ≤ PaCO_2_ < 35 mmHg, *n* = 24), green line: normal range (35 mmHg ≤ PaCO_2_ ≤ 45 mmHg, *n* = 202). Trajectories of (**a**) bias and (**b**) 1.96SD on PCO_2_. Trajectories of (**c**) bias and (**d**) 1.96SD on PO_2_. Crossing the 0 line at 2–3 min (earlier than in the other two groups), PCO_2_ biases in severe hypocapnia group was larger than those of the other two groups (**a**). This effect of hypocapnia seemed to be severity-dependent at 9 min or later. The 1.96SD of severe hypocapnia group was higher than that of the other two groups (**b, d**). The absolute values of PO_2_ bias in severe hypocapnia group were larger than those of the other two groups at 6 min or later. (**c**). **Supplementary Fig. S5.** Comparison of the time course data to evaluate effects of hypercapnia. Transcutaneous data obtained via the chest or forearm sensors were used. Average data are shown. Blue line: normal range (35 mmHg ≤ PaCO_2_ ≤ 45 mmHg, *n* = 202), red line: mild hypercapnia (45 mmHg < PaCO_2_ ≤ 50 mmHg, *n* = 26), green line: severe hypercapnia (50 mmHg < PaCO_2_, *n* = 13). Trajectories of (**a**) bias and (**b**) 1.96SD on PCO_2_. Trajectories of (**c**) bias and (**d**) 1.96SD on PO_2_. Crossing the 0 line at about 6 min (later than normal range group), PCO_2_ biases in mild hypocapnia group were smaller than those of normal range group (**a**). This delay of crossing the 0 line seemed to be severity-dependent. The 1.96SD of severe hypercapnia group on PCO_2_ was higher than that of the other two groups (**b**). The absolute values of PO_2_ bias in mild hypercapnia group were smaller than those of normal range group at 2 min or later (**c**). 1.96SD on PO_2_ seemed to be about the same among the three groups (**d**). **Supplementary Fig. S6.** Comparison of the time course data to evaluate effects of PaO_2_ levels. Transcutaneous data obtained via the chest or forearm sensors were used. Average data are shown. Blue line: hypoxemia (PaO_2_ < 80 mmHg, *n* = 158), red line: normal range (80 mmHg ≤ PaO_2_ ≤ 100 mmHg, *n* = 102), green line: hyperoxemia (100 mmHg < PaO_2_, *n* = 12). Trajectories of (**a**) bias and (**b**) 1.96SD on PCO_2_. Trajectories of (**c**) bias and (**d**) 1.96SD on PO_2_. PCO_2_ biases in hypoxemia group were smaller than those of the other two groups (**a**). The 1.96SD on PCO_2_ seemed to be about the same among the three groups (**b**). The absolute values of PO_2_ bias in hypoxemia group were smaller than those of normal range group (**c**). The absolute values of PO_2_ bias in hyperoxemia group were larger than those of normal range group (**c**). The 1.96SD of hyperoxemia group on PO_2_ was higher than that of the other two groups (**d**).

## Data Availability

The datasets used in this study are available from the corresponding author upon reasonable request.

## Abbreviations

AARC: American Association for Respiratory Care
ACO: Asthma-chronic obstructive pulmonary disease overlap
ANOVA: Analysis of variance
BGA: Blood gas analysis
COPD: Chronic obstructive pulmonary disease
IUHW: International University of Health and Welfare
PtcCO_2_: Transcutaneous PCO_2_
PtcO_2_: Transcutaneous PO_2_
SD: Standard deviation
SpO_2_: Percutaneous oxygen saturation
