# Supplementary material for: Evaluation of time courses of agreement between minutely obtained transcutaneous blood gas data and the gold standard arterial data from spontaneously breathing Asian adults, and various subgroup analyses
Source: BMC Pulm Med. 2020 May 29;20:151. doi: 10.1186/s12890-020-01184-w (PMC7257137; doi:10.1186/s12890-020-01184-w)
Supplement: Supplementary file 1 — Additional file 1: Supplementary Table S1. Comparison of subjects among the four groups of different sensor locations. Supplementary Table S2. Comparison of blood gas data among the four groups of different sensor locations. [file 12890_2020_1184_MOESM1_ESM.pdf]

Sensor location		Forearm	Chest	Earlobe	Forehead
N		143	129	17	6
Age (years old)		73.8±13.9	74.3±14.4	70.6±18.0	63.5±24.9
Gender (M/F)		84/59	84/45	12/5	4/2
Body mass index (kg/m^2)		22.2±4.0	22.0±4.6	23.0±3.1	20.7±2.6
Breakdown of subjects (biggest problem)	Healthy volunteer	4	4	1	1
	Pneumonia	42	33	3	4
	COPD (ACO, Emphysema, Bronchitis)	30 (17, 12, 1)	36 (22, 13, 1)	5 (2, 2, 1)	1 (1, 0, 0)
	Interstitial lung disease	25	16	0	0
	Asthma	16	10	3	0
	Acute bronchitis	5	8	3	0
	Lung cancer	6	4	0	0
	Sleep apnea syndrome	5	2	1	0
	Bronchiectasis	3	4	1	0
	Tumor-like shadow	3	4	1	0
	Non-COPD emphysema	2	1	0	0
	Obesity hypoventilation syndrome	0	2	0	0
	Others	4 (#1)	5 (#2)	0	0

#1: Pneumothorax, eosinophilic granulomatosis, empyema and pleuritis.
#2: Dyspnea (neurosis), bronchial foreign body, empyema, conscious loss and chronic pulmonary thromboembolism.
ACO: Asthma-chronic obstructive pulmonary disease (COPD) overlap.
COPD: Chronic obstructive pulmonary disease.
There was no significant difference in age or body mass index among the four groups (ANOVA with Tukey’s post-hoc test).

Table S1

Sensor location	**Forearm**	**Chest**	**Earlobe**	**Forehead**	**Forearm or Chest**	**All**
N	143	129	17	6	272	295
$PtcCO_2$ at 30 min. (mmHg)	45.2±5.4	45.6±8.5	46.1±4.3	49.7±6.1	45.4±7.0	45.6±6.9
$PaCO_2$ (mmHg)	40.8±5.3	40.6±8.9	38.2±5.0	40.5±6.2	40.7±7.2	40.5±7.1
$PtcO_2$ at 30 min. (mmHg)	67.8±13.0	65.6±13.4	39.9±16.9 **, ‡	26.5±13.9 **, ‡	66.8±13.3	64.4±15.8
PaO_2 (mmHg)	79.6±12.0	78.2±12.2	75.4±15.7	76.0±9.1	79.0±12.1	78.7±12.2
pH	7.43±0.03	7.43±0.05	7.44±0.04	7.43±0.04	7.43±0.04	7.43±0.04
SpO_2 (%)	96.0±2.0	95.7±2.5	94.8±6.6	96.3±1.2	95.8±2.2	95.8±2.6
Pulse (/min)	74.3±13.7	74.7±14.1	75.4±12.3	74.7±12.0	74.5±13.9	74.6±13.7

ANOVA with Tukey's post-hoc test was performed among the four sensor locations.
**: $p < 0.01$ compared with forearm. ‡: $p < 0.01$ compared with chest.
