# Supplementary figures and images for: Evaluation of time courses of agreement between minutely obtained transcutaneous blood gas data and the gold standard arterial data from spontaneously breathing Asian adults, and various subgroup analyses
Source: BMC Pulm Med. 2020 May 29;20:151. doi: 10.1186/s12890-020-01184-w (PMC7257137; doi:10.1186/s12890-020-01184-w)

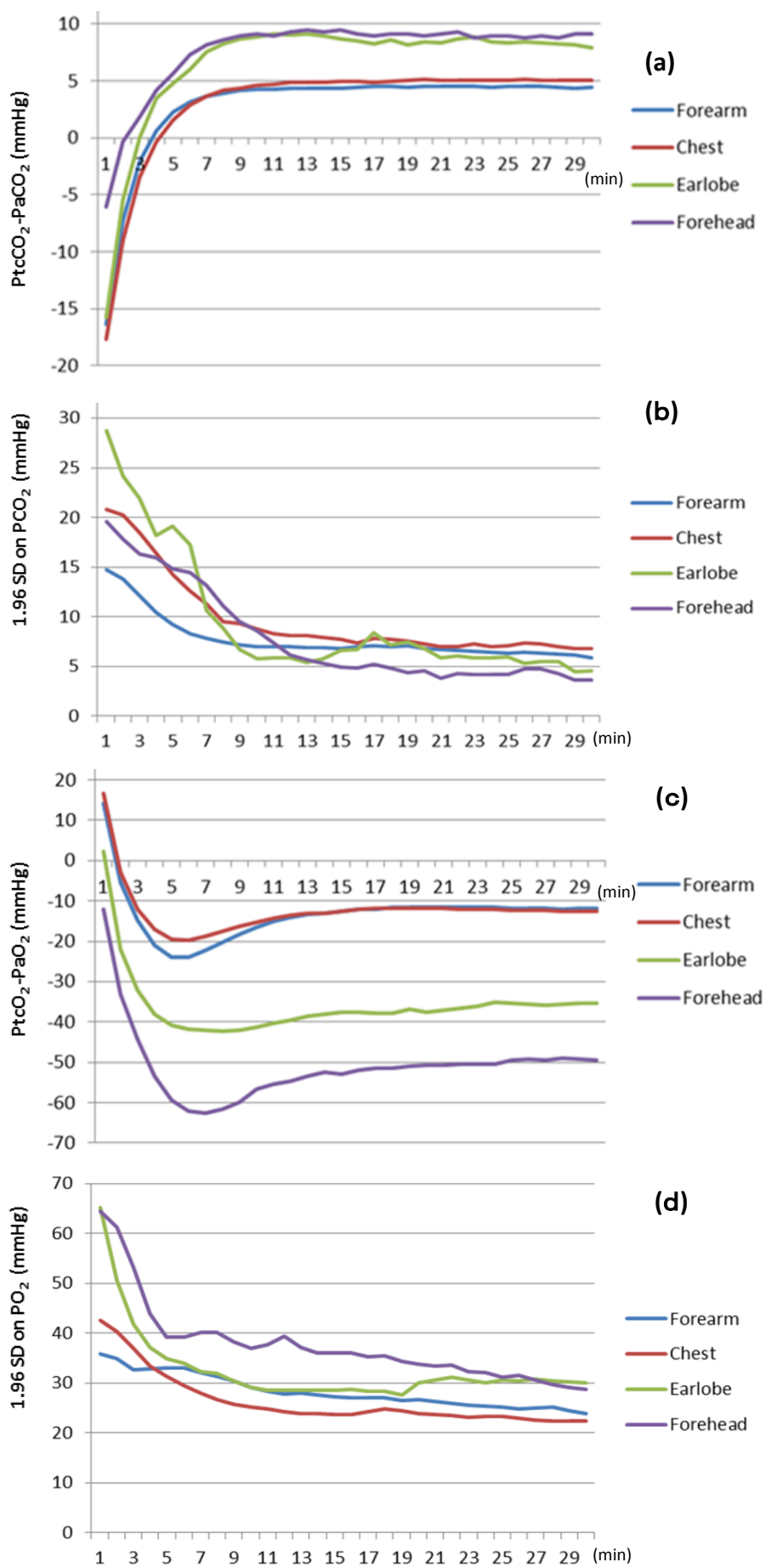

Fig. S1

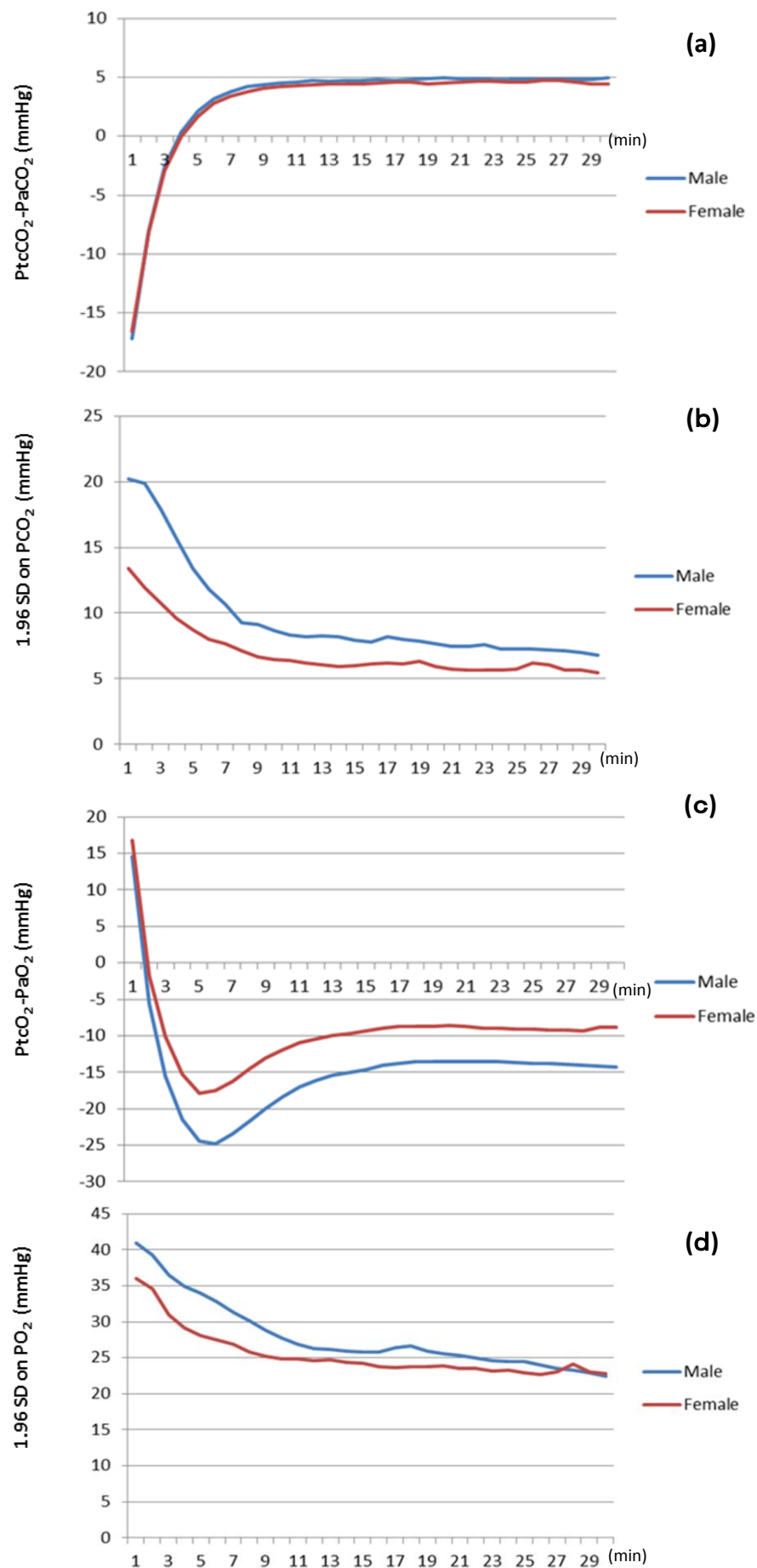

Fig. S2

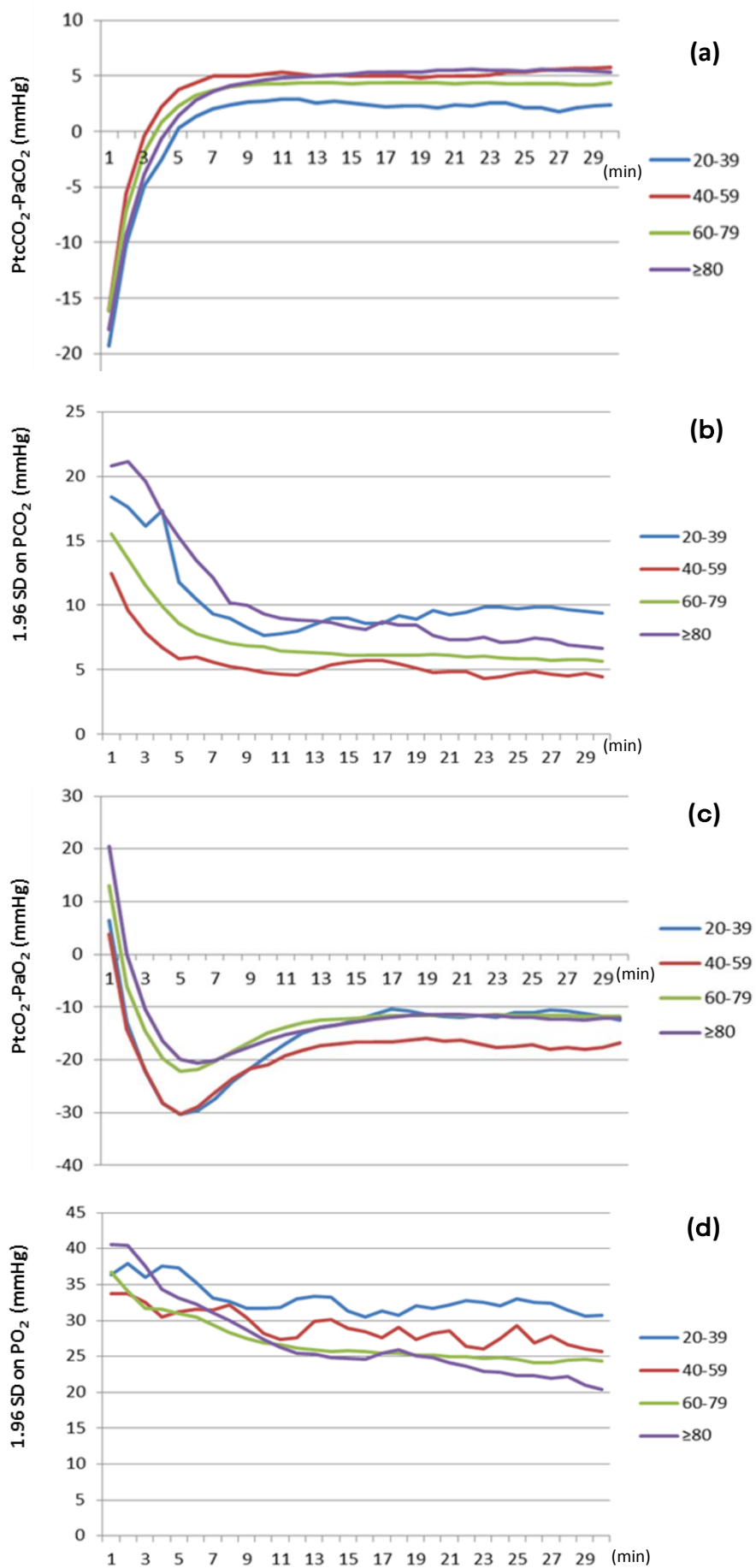

Fig. S3

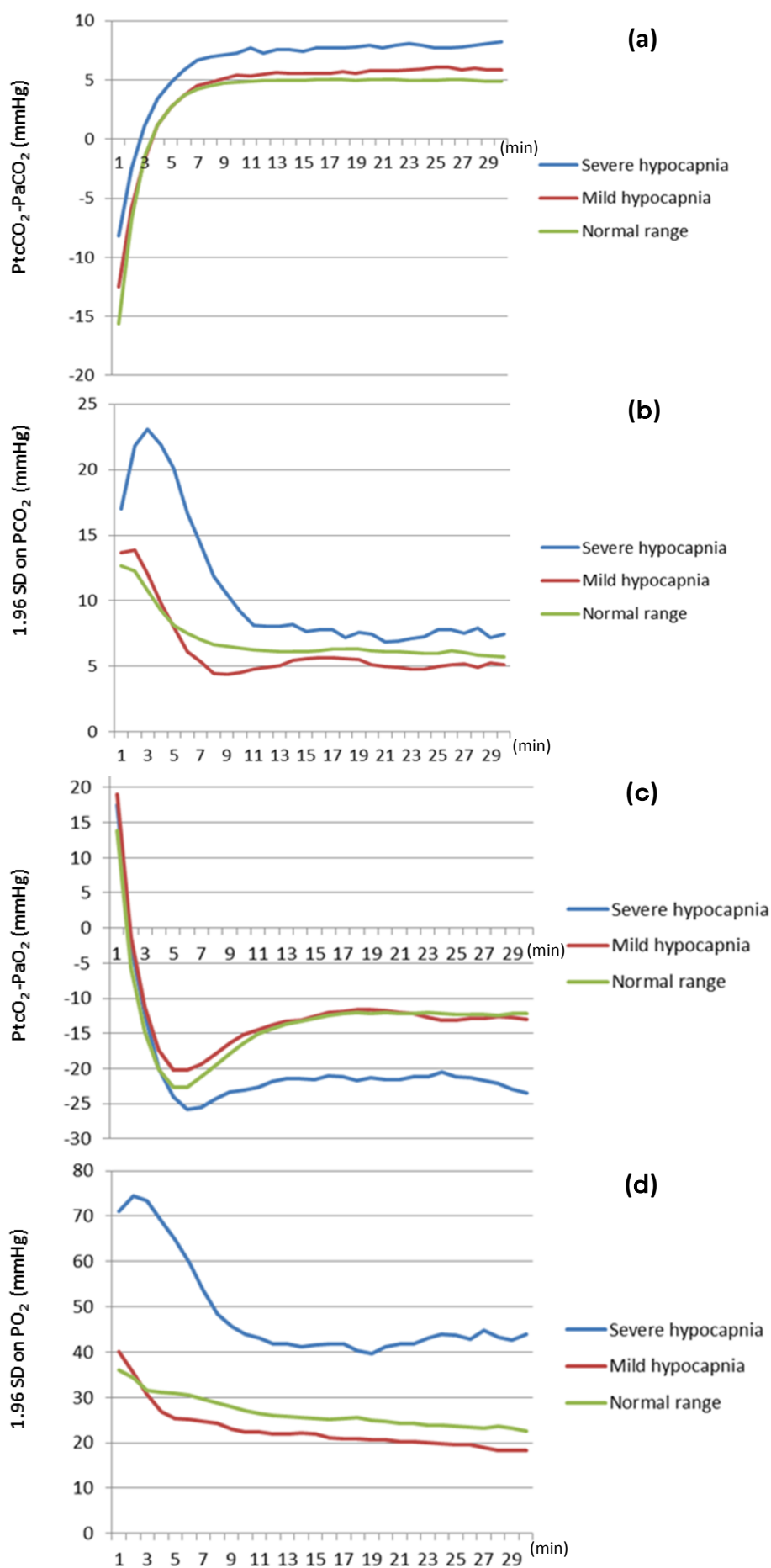

Fig. S4

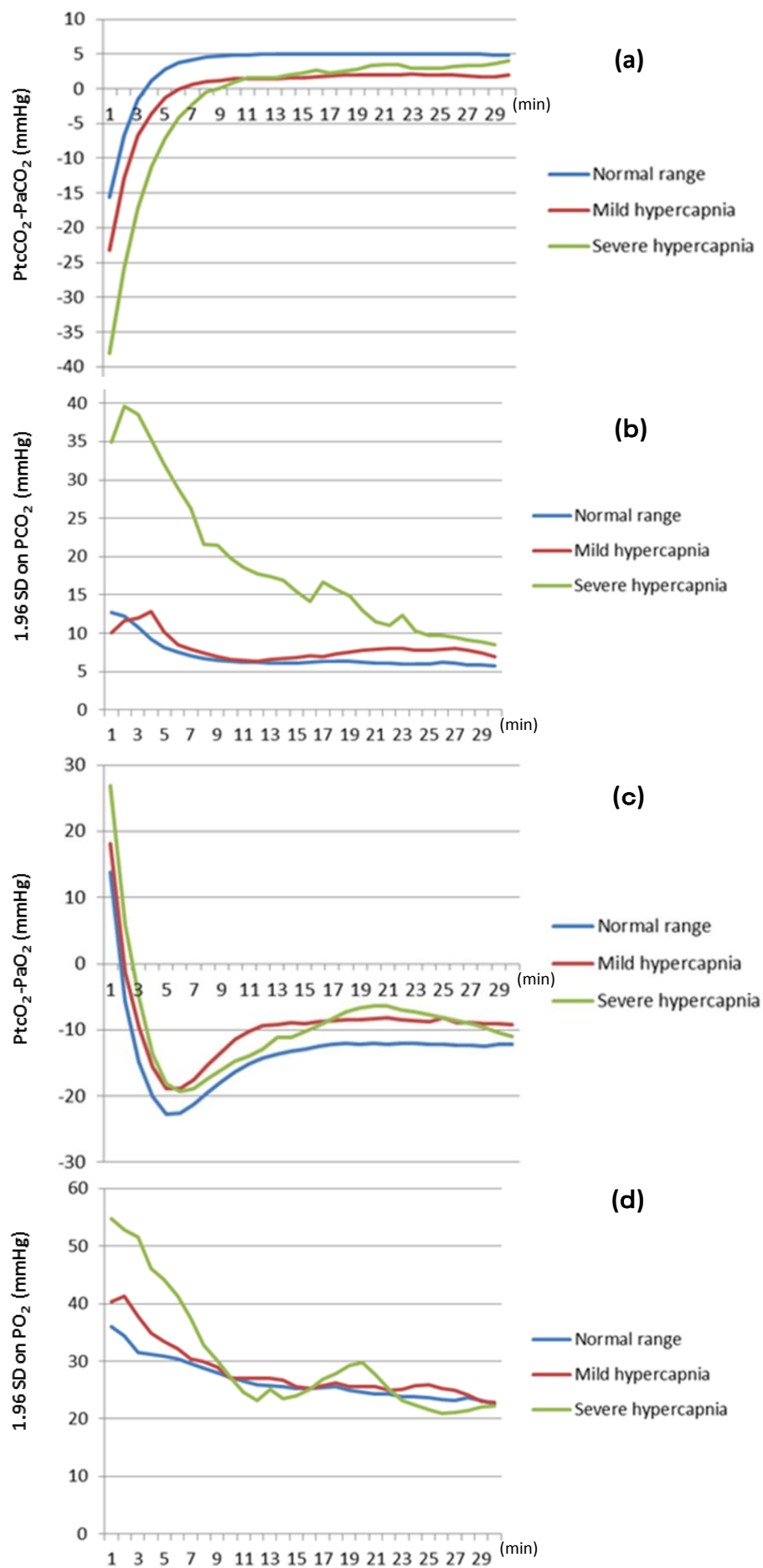

Fig. S5

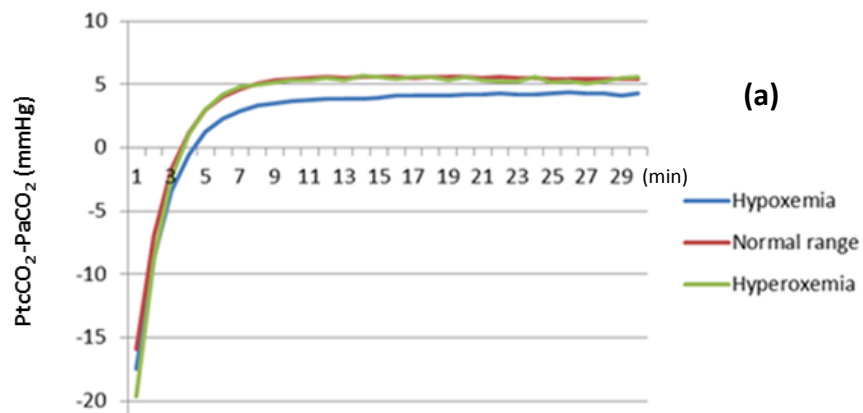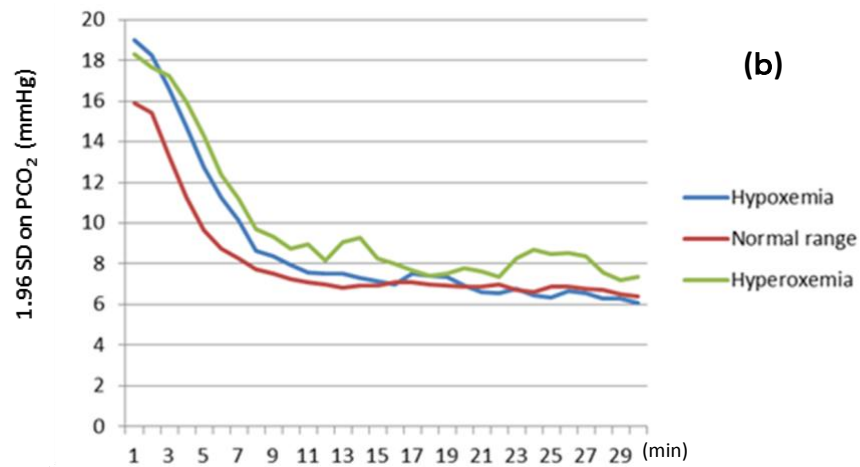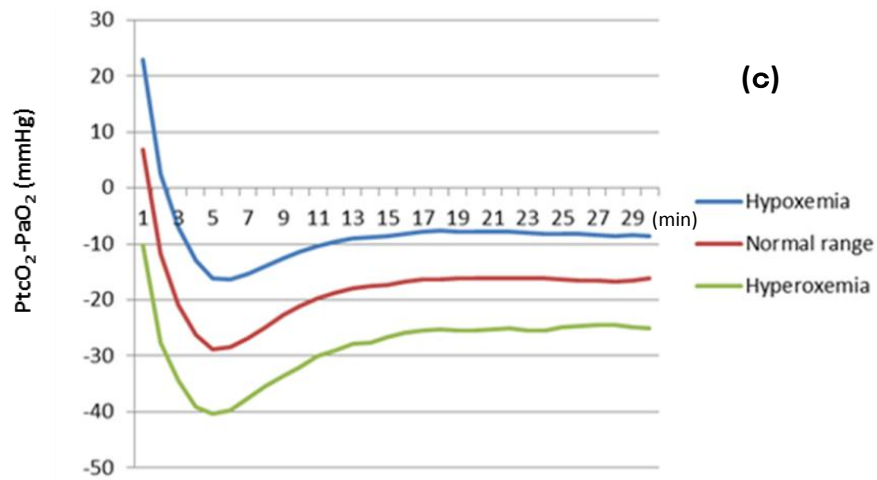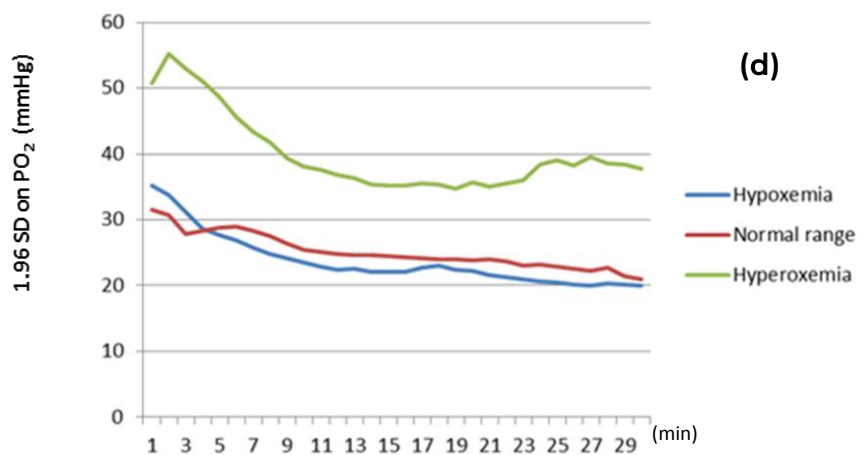

Fig. S6

Supplement: Supplementary file 2 — Additional file 2: Supplementary Fig. S1. Comparison of the time course data among the four locations of sensors. Average data are shown. Blue line: forearm (n = 143), red line: chest (n = 129), green line: earlobe (n = 17), and purple line: forehead (n = 6). Trajectories of (a) bias and (b) 1.96SD on PCO2. Trajectories of (c) bias and (d) 1.96SD on PO2. (a): Compared with the forearm or chest sensors, the earlobe or forehead sensors yielded larger bias for PCO2 at 4 min or later. (b): 1.96SD on PCO2 was similar among the four locations. (c): The forearm and chest sensors showed almost the same time course of bias for PO2. The earlobe sensor yielded larger absolute values of bias, whereas the forehead sensor yielded much larger absolute values of bias. (d): 1.96SD of the forehead sensor was larger than that of the forearm or chest sensors. Supplementary Fig. S2. Comparison of the time course data (males vs. females, n = 272). Transcutaneous data obtained via the chest or forearm sensors were used. Average data are shown. Blue line: males (n = 168), red line: females (n = 104). Trajectories of (a) bias and (b) 1.96SD on PCO2. Trajectories of (c) bias and (d) 1.96SD on PO2. PCO2 bias was similar between the two groups (a). The 1.96SD of females on PCO2 was slightly lower than male (b). The absolute values of female PO2 bias was lower than that of males (c). 1.96SD on PO2 was not affected by gender (d). Supplementary Fig. S3. Comparison of the time course data (among four age groups, n = 272). Transcutaneous data obtained via the chest or forearm sensors were used. Average data are shown. Blue line: 20–39 years (n = 11), red line: 40–59 years (n = 12), green line: 60–79 years (n = 138), purple line: ≥ 80 (n = 111). Trajectories of (a) bias and (b) 1.96SD on PCO2. Trajectories of (c) bias and (d) 1.96SD on PO2. Crossing the 0 line at approximately 5 min (later than in the other three groups), PCO2 biases in young adults (20–39 years) was slightly lower than those of the [file 12890_2020_1184_MOESM2_ESM.pdf]
